# Surgical Treatment and Targeted Therapy for a Large Metastatic Malignant Peripheral Nerve Sheath Tumor: A Case Report and Literature Review

**DOI:** 10.3390/life14121648

**Published:** 2024-12-12

**Authors:** Patryk Skórka, Dawid Kordykiewicz, Andrzej Ilków, Konrad Ptaszyński, Janusz Wójcik, Wiktoria Skórka, Małgorzata Edyta Wojtyś

**Affiliations:** 1 Department of Thoracic Surgery and Transplantation, Pomeranian Medical University in Szczecin, Alfreda Sokołowskiego 11, 70-891 Szczecin, Poland; 2 Department of General, Vascular and Oncological Surgery, Provincial Hospital, Mikołaja Kopernika, Tytusa Chałubińskiego 7, 75-581 Koszalin, Poland; 3 Department of Pathology, University Hospital of Pomeranian Medical University in Szczecin, Unii Lubelskiej 1, 71-252 Szczecin, Poland

**Keywords:** case report, NF1, MPNST1, lung metastasis

## Abstract

Neurofibromatosis type 1 (NF1) significantly increases the risk of malignant peripheral nerve sheath tumors (MPNST), a rare and aggressive malignancy for which treatment is clinically challenging. This paper presents the case of a 24-year-old male with an NF1 who developed MPNST with lung metastases. Due to the limited effectiveness of systemic therapy in the treatment of MPNST, the patient underwent radical surgical resection and radiotherapy. Pathological evaluation confirmed high-grade MPNST, and PET-CT imaging revealed further metastatic progression. The treatment results for our patient are compared with those of other patients with NF1 who also developed MPNST with lung metastases in the literature. The findings suggest the need for further research into personalized treatment strategies that may improve prognosis and overall survival in patients with NF1 and MPNST, with immunotherapy being a promising therapeutic option.

## 1. Introduction

Malignant peripheral nerve sheath tumor (MPNST) is a highly aggressive soft tissue sarcoma originating from neuroectodermal cells, specifically Schwann cells associated with the peripheral or cranial nerves. The broader category of nerve sheath tumors also includes schwannoma and neurofibroma. MPNST often develops de novo or through the malignant transformation of benign nerve tumors, such as neurofibroma [1,2]. The disease is exceptionally rare, with an incidence rate of 0.001% in the general population and representing approximately 5% of all soft tissue sarcomas [3,4,5]. MPNST is most frequently associated with neurofibromatosis type 1 (NF1), and individuals affected by NF1 have an estimated lifetime risk of developing MPNST of 8–13% [6]. MPNST predominantly manifests in adults between 20 and 50 years of age, but it can also occur in children, primarily in association with NF1. Compared to the general population, individuals with NF1 have a predisposition to develop various malignancies, including MPNST, at significantly younger ages. The youngest reported case involved a 20-month-old toddler [7]. MPNSTs commonly occur in the peripheral nerves of the trunk and extremities, but they can also involve the cranial nerves. The tumors may affect various nerve structures, including the peripheral nerves, brachial and sacral plexuses, and spinal nerve roots. Analysis of 3267 cases of MPNST documented in the Surveillance, Epidemiology, and End Results (SEER) database from 1973 to 2013 revealed that 40.0% (*n* = 1307) were found in the spinal cord, 31.3% (*n* = 1022) in the extremities, 13.7% (*n* = 449) in the head and neck region, 6.2% (*n* = 203) in unspecified locations, 5.1% (*n* = 167) were located in the skull, and 3.6% (*n* = 119) in the spine [8]. These tumors tend to recur locally, even after radical surgical resection, and can metastasize to the lungs, liver, lymph nodes, and bones. Due to their aggressive nature, MPNSTs are associated with poor overall survival rates (OS), with 5-year survival rates of 40–50% [2,9]. The prognosis is even worse for patients with metastatic MPNST [10]. Akshintala et al. reported a baseline progression-free survival (PFS) of only 1.77 months for patients with recurrent, unresectable, or metastatic disease [11]. Several independent factors correlate with reduced survival, including advanced age, male sex, black race, absence of surgical resection, incomplete tumor removal, larger tumor size, higher tumor grade, and tumor localization in the head and neck region [8]. In addition, patients with MPNST have a significantly higher incidence of various secondary malignancies compared to the general population. In females, secondary cancers frequently involve the breast, lung, skin, and soft tissues, whereas males more commonly develop secondary myeloid and skin cancers [12].

MPNST presents substantial clinical challenges due to the aggressive nature of the disease. Given the tumor’s limited responsiveness to systemic therapies, wide-margin surgical resection remains the cornerstone of treatment. An additional challenge in developing an optimal treatment strategy is the scarcity of published cases in the literature. In this article, we attempted to compare the diagnostics and treatment outcomes of our case and similar cases of NF1 patients who developed MPNST with lung metastases published in the literature.

## 2. Detailed Case Description

**Figure 1:**
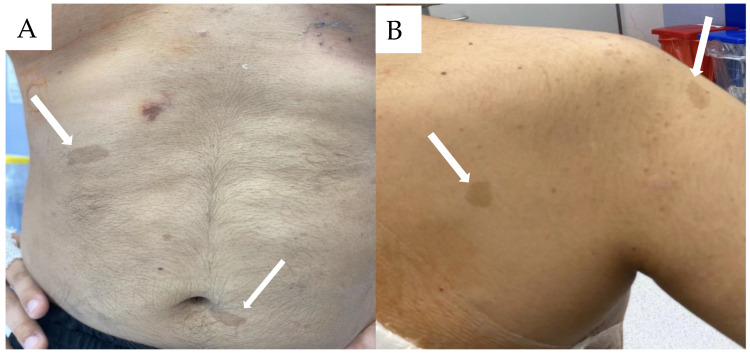
Patient’s current café au lait spot in the trunk (**A**,**B**).

A 24-year-old male non-smoker was admitted to the Department of Thoracic Surgery and Transplantation, Pomeranian Medical University in Szczecin, in October 2024 due to a metastatic tumor of the right side of the thoracic cavity emerging from the inferior mediastinum. The patient’s medical history indicated the initial appearance of a café au lait spot in the left popliteal fossa at the age of 5 (Figure 1).

Four years later, a correction with anterior L1–L3 spondylodesis using the Baguera Oblique Block (B.O.B) technique was performed due to left lumbar systemic scoliosis. One year later, an extension of the spondylodesis to Th11 was performed along with partial resection of an unknown mass in the retroperitoneal space of the left lumbar region. Complete resection of the mass was not possible due to limited access to the tumor. Histopathological examination confirmed a neurofibroma. A mandibular epidermal cyst was excised 11 years later. Based on the clinical symptoms of a 21-year-old patient, next-generation sequencing (NGS) analysis of the NF1, NF2, SMARCB1, and SPRED gene sequences was performed, which revealed the presence of the c.1381C>T variant in one allele of the NF1 gene. The diagnosis was supported by clinical symptoms observed over many years. In October 2023, a core needle biopsy was performed on a giant sacrococcygeal tumor in the left lumbar region. The tumor was a malignant spindle cell neoplasm containing pleomorphic cells, necrotic areas, and pathological mitotic figures. The result was consistent with an MPNST with a high degree of malignancy. In addition, an MRI indicated infiltration of the intervertebral foramina by the tumor. The patient was offered palliative chemotherapy, which the patient rejected. In December 2023, the described tumor was resected, it disintegrated, but the surgical margin was negative. Histopathological examination revealed fragments of MPNST tissue containing cells with pleomorphic nuclei and areas of necrosis. Additionally, during the operation, the surgeon identified five distinct tumors in the retroperitoneal pelvic space, which was later confirmed by computed tomography (CT) imaging. One month after surgery, FISH analysis identified the MDM2 gene in the patient, confirming its amplification.

**Figure 2:**
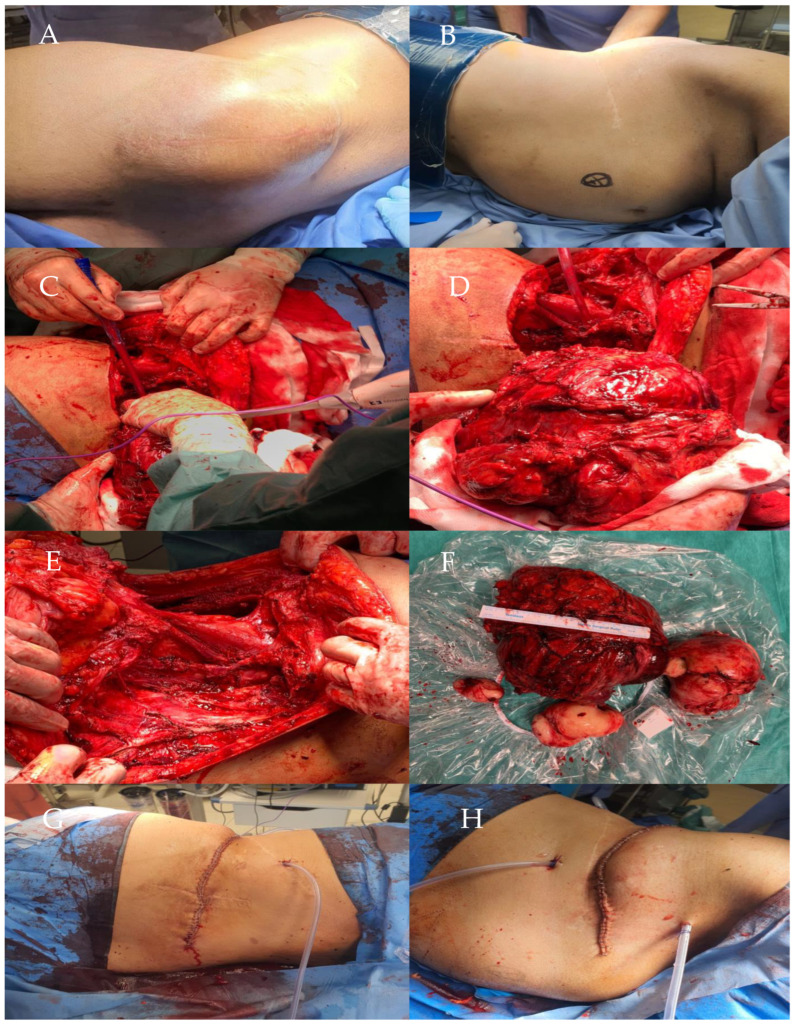
Preoperative image showing a tumor in the left retroperitoneal space infiltrating adjacent structure (**A**). Tumor in the left lumbar region visualized during surgery, showing extensive neoplastic tissue (**B**). Postoperative image showing significantly reduced tumor masses in the lumbar region after the initial resection (**C**). Image confirming the presence of a new tumor in the left retroperitoneal space, requiring further surgical intervention (**D**). Cross-sectional images of removed tumors from the left lumbar region and retroperitoneal space (**E**,**F**). Post-operative wound with the drainage system (**G**,**H**).

Five months later, a laparotomy was performed to resect five tumors in the left retroperitoneal space and left lumbar region due to extensive malignant tumor recurrence during the course of an abortive form of neurofibromatosis (Figure 2).

**Figure 3:**
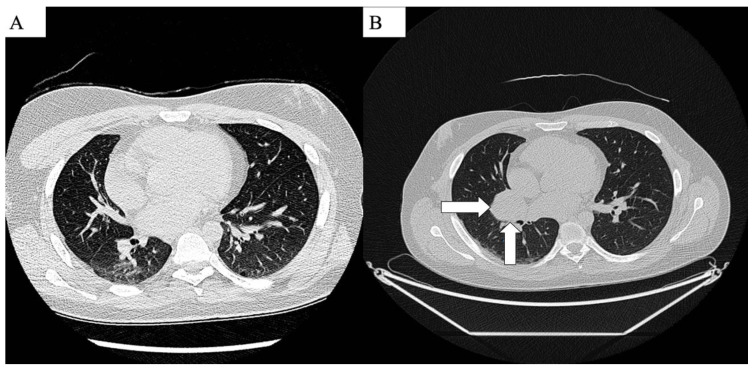
PET/CT axial plane. (**A**) Showing no lung lesions April 2024. (**B**) Lung metastasis July 2024 (white arrows).

Histopathological examination of these tumors confirmed the diagnosis of a malignant peripheral nerve sheath tumor (MPNST) in the left lumbar region, measuring 24.0 × 21.0 × 12.0 cm. Additionally, four neurofibromas were identified: one in the left paravertebral retroperitoneal region measuring 12.5 × 10.5 × 8.0 cm, another in the lesser pelvis of the sacrum measuring 9.5 × 6.0 × 5.5 cm, a satellite tumor in the lumbar region measuring 5.5 × 4.0 × 3.0 cm, and a paraspinal satellite tumor measuring 5.0 × 4.0 × 3.2 cm. Positron emission tomography (PET/CT) performed after surgery showed a metabolically active tumor medially from the lower pole of the left kidney retracting the left ureter, size 46 × 38 × 43 mm, SUVmax 8.8, and a metabolically active focus in the paraspinal muscles at Th12/L1 level, size 20 × 23 mm, SUVmax 6.6. The patient then underwent 2 months of radiotherapy treatment, complicated by sciatic nerve palsy on the left side. A subsequent PET/CT showed a tumor in the retroperitoneal space, located in the surgical locus of the rectus dorsi muscle. The tumor had features of malignancy with metastasis to the spine on the left side and with metastasis to the right lung. Chest PET/CT showed a tumor in the interlobar oblique fissure measuring 45 mm and two smaller nodules of 10 and 8 mm in the lower part of the upper lobe of the right lung within segment III (Figure 3).

**Figure 4:**
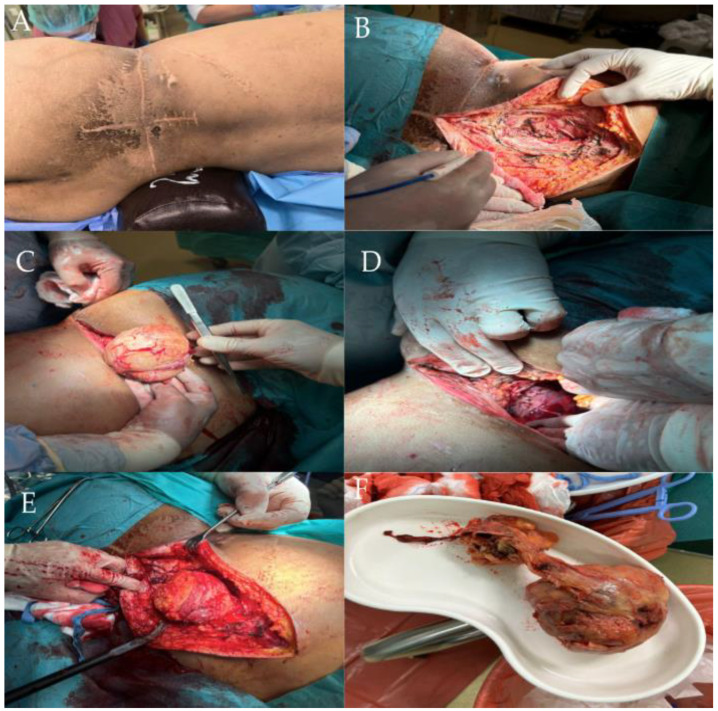
Intraoperative view showing the left lateral retroperitoneal tumor during exposure. The tumor is partially visible within the surgical field (**A**). Close-up of the left retroperitoneal tumor during resection, highlighting its dense and fibrotic structure (**B**). Advanced stage of dissection in the left retroperitoneal region, revealing tumor adherence to adjacent tissues (**C**). Completely resected tumor mass from the left retroperitoneal space, with irregular margins visible (**D**). Post-resection surgical field showing the cavity left by the removed tumor (**E**). Resected tumor mass (**F**).

Two months after radiotherapy, the patient was admitted to the Department of Surgical Oncology, complaining of a cough, shortness of breath, tachycardia, fever, and right shoulder pain. The symptoms did not resolve after surgery. A tumor resection was performed from the left lateral retroperitoneal space measuring 10.0 × 10.0 × 8.0 cm, along with another tissue fragment measuring 12.0 × 3.0 × 0.5 cm, and distally measuring 5.0 × 7.0 cm (Figure 4).

**Figure 5:**
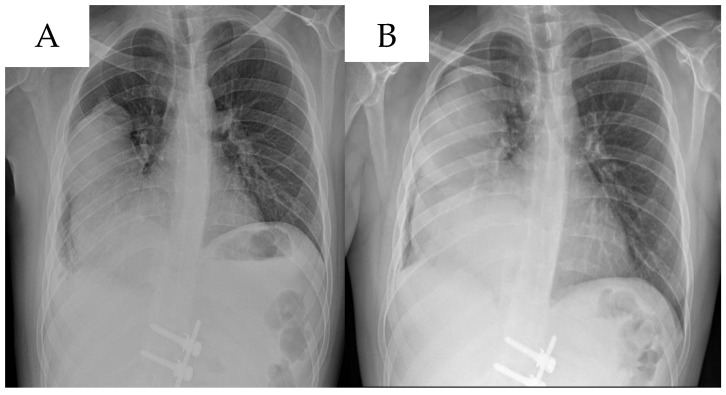
Chest X-Rays performed a one-month interval showing progression of lung metastasis. (**A**,**B**).

Histopathological examination once again indicated MPNST. Two and three months after the last intervention, X-Rays were taken documenting lung tumor progression (Figure 5).

**Figure 6:**
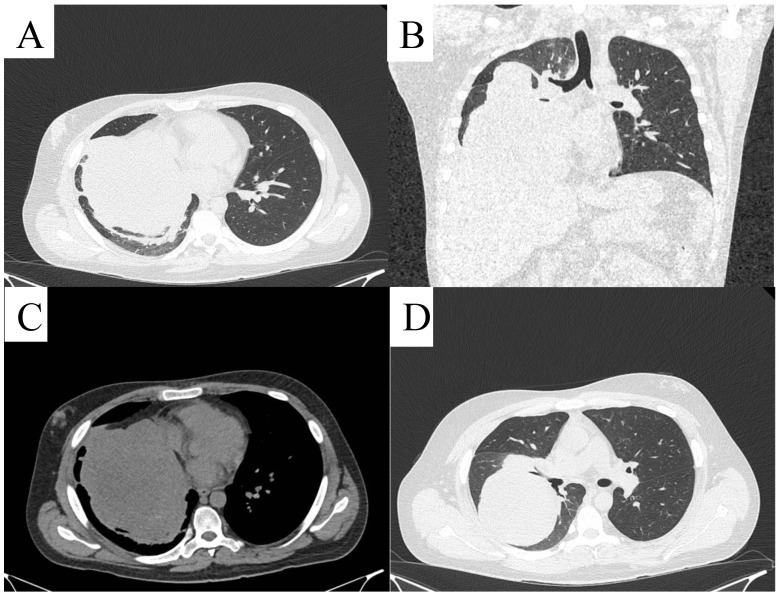
Chest CT: axial plane (**A**,**C**,**D**), coronal plane (**B**) heterogeneous density lesion of the right lung lobule with displacement of mediastinal structures.

One week before admission to the hospital, a chest CT showed extensive tumors of the right lung involving all lobes and measuring 140 × 119 × 183 mm. The tumor was adjacent to the pericardium, diaphragm, pleura, right pulmonary artery, and intermediate bronchus. The periphery of the tumor showed atelectatic changes (Figure 6).

**Figure 7:**
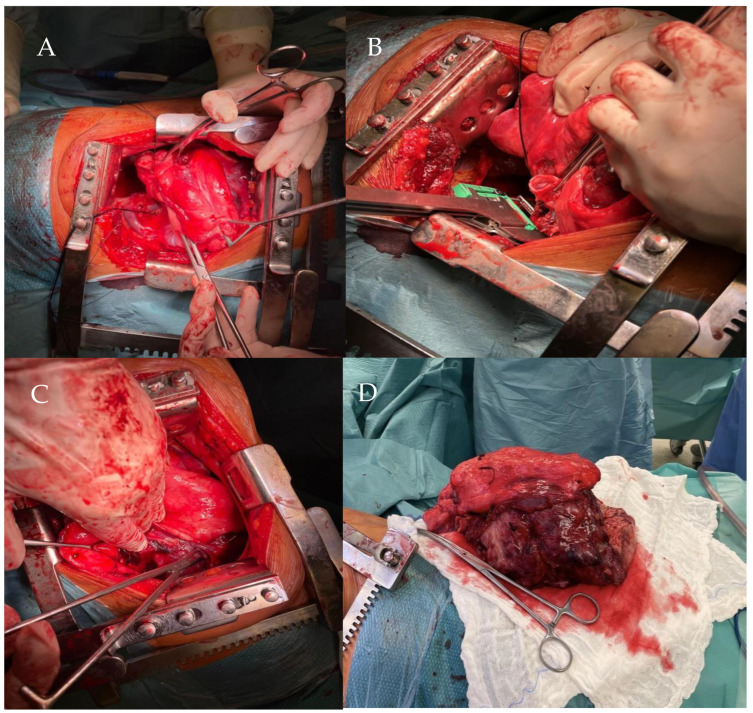
The initial phase of right thoracotomy reveals the metastatic MPNST mass in the thoracic cavity (**A**). The tumor during partial separation from the surrounding tissues, demonstrating anatomical infiltration (**B**). Post-thoracotomy view showing the space left after the complete resection of the metastatic tumor from the right lung (**C**). Final operative field view after the complete tumor resection, illustrating the area cleared of the mass (**D**).

Following this diagnosis, the patient was admitted to the Department of Thoracic Surgery and Transplantation, presenting with a chronic cough accompanied by expectoration of white sputum, dyspnea, heartburn, intermittent tachycardia, and right shoulder pain. A right-sided pneumonectomy followed by a mediastinal lymphadenectomy was performed. A tumor measuring 220 × 130 mm was identified. Subsequently, the right branch of the pulmonary artery was isolated, ligated, and transected. The right main bronchus was severed with a stapler, after which the esophagus was dissected from the tumor. The pericardial sac was opened from the front and the isolated superior pulmonary vein was fixed with a stapler. The inferior pulmonary vein was severed with a stapler and the tumor was dissected from the mediastinum together with the severed phrenic nerve. The tumor was detached from the diaphragm and removed. In addition, lymph nodes 4R, 7, 10R and the diaphragmatic infiltrate through the tumor, and a fragment of the parietal pleura was removed. (Figure 7). After the surgery, the patient’s well-being improved, with no shortness of breath or coughing attacks. The pain in the right shoulder and arm subsided. There was only slight irritation in the throat.

**Figure 8:**
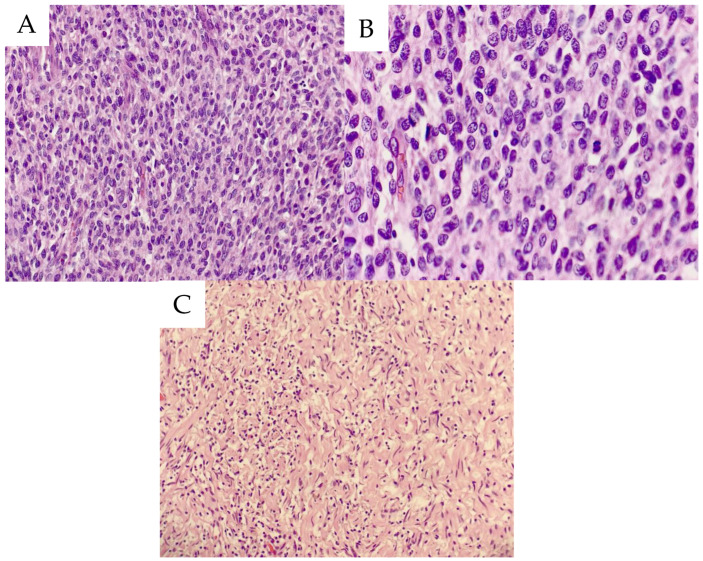
Histopathological specimens (representative hematoxylin–eosin stained histological images): (**A**) Spindle- and oval-shaped cells display a vague fascicular growth pattern with some perivascular accentuation. A moderate number of mitotic figures are present (original magnification ×200). (**B**) A high-power view of the spindle- and oval-shaped cells reveals a vague fascicular growth pattern (original magnification ×400). (**C**) A paratracheal nodule shows histological features of neurofibroma (original magnification ×200).

A gross examination of the specimen indicated that the right lung was 80% filled by a tumor with the largest dimension of 220 mm. Histopathology showed a spindle cell, partially pleomorphic malignant neoplasm with prominent necrosis, high mitotic activity, and perivascular accentuation, consistent with a metastatic MPNST. Additionally, a paratracheal nodule was identified with histological features of a neurofibroma. The tumor locally crossed the pleura and the surgical margin, which was confirmed microscopically. (Figure 8). After removing the metastases, the patient qualified for treatment with Trametinib, an oral anticancer drug that inhibits mitogen-activated protein kinase 1/2 (MEK1/2).

## 3. Discussion

MPNST typically presents as an enlarged soft tissue mass originating from a peripheral nerve. In this case, the patient’s initial diagnosis was NF1. However, the disease later presented as MPNST localized to the lumbar region, followed by MPNST with metastasis to the right lung parenchyma. Additionally, a nodule resembling a neurofibroma was identified in the paratracheal area. Upon reviewing the available literature in medical databases, we found only a few cases with similar specificity, five of which were treated surgically (Table 1).

The primary diagnostic modalities for detecting MPNST are CT and MRI, and they were used in all the presented cases. These imaging techniques are crucial for assessing the tumor’s location, size, and potential metastatic spread. Notably, the tumor is enhanced after contrast administration [16]. Moreover, in patients with neurofibromatosis type 1 (NF1), MRI employing apparent diffusion coefficient (ADC) values derived from diffusion-weighted imaging (DWI) has proven effective for distinguishing between benign and malignant peripheral nerve sheath tumors [20,21,22]. Furthermore, Yun et al. reported that the absence of the split fat sign and specific mean apparent diffusion coefficient (ADC) values were identified as solid imaging indicators of malignancy in MPNSTs during MRI diagnostics [23]. These findings align with previously mentioned studies and highlight the importance of advanced imaging techniques in clinical practice. Furthermore, the diagnostic methods that would be useful in our patient’s diagnostic process are three-dimensional high-resolution ultrasound (3D HRUS) and magnetic resonance microscopy (MRM). MRM is particularly beneficial in the preoperative planning process showing a detailed reconstruction of the nerves and fascicles indicating the connections between them. In the case of MPNST, it allows accurate estimation of nerve volumes and surgical planning. Three-dimensional HRUS, due to the limitations of this method, can be used as an adjunctive tool in the intraoperative setting when MRM availability is limited [24].

Patients with von Recklinghausen syndrome (NF1) have a 113 times higher risk of developing MPNST compared to the general population. Distinguishing between benign NF1 and MPNST requires advanced diagnostic techniques, as in our case PET-CT to detect malignant transformation and metastasis. Therefore, patients with NF1 and MPNST present a major diagnostic challenge. Additionally, 10% of NF1 patients struggle with orthopedic problems such as scoliosis [25]. This also affected our case, as the patient underwent two surgeries for advanced scoliosis.

Furthermore, 18F-fluorodeoxyglucose (FDG) uptake provides valuable insights by visualizing metabolic activity, which aids in assessing tumor aggressiveness. Benign lesions typically exhibit little to no FDG uptake, whereas malignant tumors tend to show significantly higher FDG accumulation. The case described here also underwent PET, which identified a malignant tumor with metastasis to the spine and right lung.

This information can significantly enhance the precision of tissue targeting and sampling during biopsies, especially when other diagnostic modalities fail to identify abnormalities. Brahmi et al. demonstrated that PET/CT-guided percutaneous biopsy has a high diagnostic accuracy of 96% [26]. This underscores the potential of PET/CT as an effective tool for accurately identifying abnormalities in challenging clinical scenarios where other imaging modalities may be less conclusive. Biopsy procedures carry inherent risks, particularly regarding complications such as pain or nerve damage. However, despite these concerns, biopsies remain valuable diagnostic tools for MPNST, showing a high correlation with histological findings obtained from surgical resection samples [26,27]. Core needle biopsy remains the gold standard for confirming the histopathological diagnosis of MPNST. In our patient, this type of biopsy was performed for a sacrococcygeal tumor combined with a comprehensive clinical evaluation and imaging techniques (e.g., MRI). This method ensures precise tumor characterization, critical for tailoring appropriate treatment strategies.

Monitoring plays a vital role in the management of patients with NF1 due to their heightened tumorigenic risk. Continuous surveillance is necessary to assess the total volume and number of neurofibromas, as well as promptly identify any emerging symptoms that may signal progression. Because of a substantial correlation between their quantity, the total volume of neurofibromas, and the likelihood of transformation into MPNST, prompt identification is crucial [28]. This proactive approach enables early intervention, which is critical to mitigating complications. Regular monitoring is also essential to assess the progression of structural infiltration by the MPNST, as evidenced by the high rate of tumor growth in the lung of our patient. The management of patients with MPNST requires the cooperation of a multidisciplinary team, including specialists in oncology, radiology, surgery and pathology [29].

In patients with NF1 who have plexiform neurofibromas, PET-CT has proven to be a valuable tool for monitoring and detecting malignant lesions. Studies have demonstrated that PET-CT has 100% sensitivity in identifying malignant transformation, even in asymptomatic individuals [30]. Key risk factors for MPNST development include the presence of plexiform neurofibromas, prior exposure to radiotherapy, and significant NF1 gene mutations [10,25,31,32,33,34]. In the presented case, the patient exhibited a c.1381C>T variant in one allele of the NF1 gene. Additionally, an MDM2 gene amplification was later identified. The MDM2 protein plays a critical role in oncogenesis by binding to the p53 tumor-suppressor protein, leading to its degradation and inhibiting its tumor-suppressive functions. The amplification of the MDM2 gene results in an overproduction of the MDM2 protein, which further disrupts p53 activity, thereby facilitating tumor progression and contributing to the malignancy of the tumor [35,36]. Awareness of these risk factors is essential for timely and effective management. A promising new diagnostic approach for distinguishing benign lesions from MPNSTs involves analyzing cell-free plasma DNA (cfDNA). Recent studies, including those by Szymanski et al., have demonstrated that the tumor fraction derived from cfDNA fragments is significantly higher in patients with MPNSTs compared to those with benign lesions or healthy individuals [37]. This finding suggests that cfDNA could serve as a non-invasive biomarker for the early detection and monitoring of MPNST, particularly in high-risk populations such as those with NF1.

Symptoms of MPNSTs depend mainly on the location of the tumor and may arise several months before the tumor becomes clinically detectable, particularly in anatomical locations that are difficult to examine, such as the retroperitoneal space. Early signs can be subtle and often include pain, neurological deficits, or other non-specific symptoms, which may lead to a delayed diagnosis. MPNSTs can manifest with local or radicular pain, paresthesia, or paraparesis, though neurological deficits are relatively uncommon. Baehring et al. reported that a painless mass was the most frequently observed non-neurological symptom in patients [5]. In contrast, in the case we described, the patient reported pain related to the described mass. In the other analyzed cases, symptoms were rarely reported; however, pain was also the most described, noted in 2 out of 12 cases [14,16]. The disease may remain clinically silent for an extended duration, with initial symptoms often emerging as respiratory complaints, particularly when lung metastases are present. In the presented case, the patient experienced shortness of breath, a symptom not commonly reported in other studies. In addition, the patient complained of heartburn, dyspnea, intermittent tachycardia, right shoulder pain, and expectoration of white sputum. This discrepancy may be due to regular diagnostic follow-ups enabling early detection of metastatic changes, or it could result from a lack of detailed symptom documentation in case reports. Further research is necessary to understand these variations. Histologically, MPNSTs typically exhibit a fascicular growth pattern composed of hyperchromatic spindle cells with elongated nuclei and regions of perivascular hypercellularity. High mitotic activity and areas of geographic necrosis are also frequently observed [4,31]. Some rare variants of MPNST demonstrate unusual histological features, including epithelioid, glandular, primitive, pleomorphic, myxoid, Schwannian, or rhabdomyosarcomatous differentiation [38,39]. The tumor in our patient consisted of a malignant spindle cell neoplasm and the presence of pleomorphic cells, areas of necrosis, and pathological mitotic figures. These findings are consistent with the case reports from the literature.

The most important surgical method for MPNST is surgical treatment. Surgical intervention in our patient, including multiple resections and radical lung surgery, was the mainstay of treatment due to the patient’s lack of consent to systemic therapy such as chemotherapy. Furthermore, the aim of such treatment is complete tumor resection achieving negative margins. The status of the margins, the size of the tumor and its location are the most common prognostic factors for MPNST [13]. The choice of treatment depends on the location and size of the tumor. As reported by Cuhna et al. in a study group of five patients with NF1 and lung metastases, only two underwent resection. Radical surgical resection in such a low percentage of patients may have been due to the advanced stage of the disease and the location of the tumor preventing effective surgical therapy. The OS was 40.80 months and 14.70 months, respectively. One patient was treated with radiotherapy achieving a very low OS [17]. Other authors have reported a high success rate for chiropractic resection achieving complete tumor resection in 69% of cases [5].

In cases in which surgical treatment is no longer a viable option, further interventions such as chemotherapy and radiotherapy become critical, particularly in the context of local recurrence or metastasis following surgery. For tumors that are non-resectable, exhibit mass effect, or are larger than 5 cm in size, neoadjuvant treatment should be considered to decrease the tumor volume and enhance the possibility of successful surgical resection. However, there is insufficient evidence to support the effectiveness of this treatment. Baehring et al. reported that adjuvant or neoadjuvant radiation therapy decreased the risk of death [5]. Conversely, Kahn et al. reported that radiation therapy did not significantly influence OS as a prognostic factor [40]. Although radiotherapy has not been proven definitively to improve long-term survival in patients with MPNST, it is often utilized in a manner consistent with the treatment of other soft tissue sarcomas. Radiotherapy can enhance local tumor control and potentially delay recurrence. Adjuvant radiotherapy is generally recommended for intermediate- to high-grade tumors and may also be considered for low-grade tumors following a marginal excision to minimize the risk of local relapse [41]. The patient in the case we described refused chemotherapy; therefore, surgical resection was the method of choice.

MPNSTs exhibit poor responsiveness to chemotherapy, thus, systemic chemotherapy often is reserved for advanced cases. There is no proven superiority of one chemotherapeutic agent over another for treatment. Typically, drugs used in the regimens are doxorubicin and ifosfamide. One patient received two cycles of neoadjuvant chemotherapy ifosfamide, etoposide, and carboplatin (ICE) to achieve tumor shrinkage. This was followed by complete tumor resection and 12 cycles of adjuvant chemotherapy with the ICE regimen. The patient has been disease-free for 12 years [13].

Other agents, including etoposide and epirubicin, have also been used either alone or in combination. The choice of a specific chemotherapy protocol often depends on the patient’s condition, the extent of the disease, and the preference of the treating physician, with a focus on controlling tumor growth and minimizing toxicity. Doxorubicin-based cytotoxic chemotherapy remains the standard treatment for unresectable or metastatic MPNSTs [6]. The addition of ifosfamide to the treatment regimen has also been studied, but it did not result in a significant improvement in survival rates [42]. However, patients experienced increased complications, particularly due to heightened myelosuppression. Chemotherapy does not significantly improve survival rates and is typically reserved for advanced cases with a poor prognosis. The 5-year OS remain low, as the disease is marked by rapid progression and high mortality. Consequently, fewer than 40% of patients with unresectable or metastatic tumors survive beyond 1 year after diagnosis [5]. Such treatment is generally not curative but may be beneficial as part of palliative care. It can help in symptom relief and prolongation of local disease control. In addition, it may be used in the preoperative setting to downstage tumors initially considered unresectable, thereby improving the potential for successful surgical resection. However, the OS advantage observed with this approach is not significant. The therapeutic effect may contribute to localized disease control, but the evidence does not support a substantial impact on long-term survival outcomes [41,43].

Thus far, studies on the search for a targeted therapy that would prove to be a clearly superior treatment regimen for MPNST are still in progress. In the described case, following pneumonectomy, the patient was qualified for treatment with the (MEK1/MEK2) pathway inhibitor, trametinib. MPNST with NF1 mutation leads to the promotion of the Rapidly Accelerated Fibrosarcoma/Mitogen-Activated Protein Kinase/Extracellular Signal (RAS/RAF/MEK/ERK) pathway [44]. NF1 mutation results in loss of function of neurofibromin, which plays an important role in RAS regulation. Disruption of the RAS pathway activates the RAF/MEK/ERK kinase leading to tumor cell proliferation [45]. Therefore, the use of MEK inhibitors may also have great therapeutic potential in our patients. Moreover, trametinib in a patient with NF1 and MPNST showed a significantly higher treatment efficacy with remission lasting 10 months than classical systemic therapies such as ifosfamide with etoposide. In addition, the patient achieved an effective radiological response to trametinib therapy in all disease foci [46]. Inhibition of the described pathway leads to a reduction in tumor proliferation. The use of MEK inhibitors in monotherapy has some limitations, as it leads to the enhancement of Phosphatidylinositol 3-Kinase/Protein Kinase B/Mechanistic Target of Rapamycin signaling (PI3K/AKT/mTOR) signaling. Activation of alternative kinases in this PI3K/mTOR shuttle can already be observed after 2–21 days of therapy [47]. Therefore, combination therapies with Mesenchymal–Epithelial Transition Factor (MET) inhibitors may be a better option, but the current treatment strategy for our patient is based on monotherapy [44]. However, NF1-dependent MPNST is characterized by high signaling variability, which poses a major challenge in the future follow-up of our patient. Another novel treatment technique may be the use of cold atmospheric plasma (CAP). This is a new therapeutic option for NF1 patients with MPNST that may also complement existing modalities [48].

Tumors in patients with NF1 exhibit an upregulation of the PD-L1. This increased PD-L1 expression contributes to the tumor’s ability to evade immune surveillance by inhibiting tumor-specific immune responses, which would otherwise induce apoptosis in cancer cells [44]. Inhibiting the interaction between PD-L1 and its receptor PD-1 reactivates pre-existing antitumor immunity by restoring the ability of T cells to recognize and attack cancer cells [45]. Davis et al. reported that treatment with pembrolizumab in a patient with metastatic MPNST resulted in a complete metabolic response after four cycles of therapy [49]. Comparable efficacy was also reported by Larson et al., who documented a case achieving complete remission by the sixth cycle of treatment. Treatment with pembrolizumab was implemented after resection of the chest wall tumor following an unsuccessful treatment cycle with epirubicin and ifosfamide. This completely different treatment regimen from our patient was due to different mutations. [19]. This was made possible through molecular profiling, which revealed 70% PD-L1 expression using immunohistochemistry. Additionally, pathogenic mutations were identified in several key genes, including ARID1A, CDKN2A, KMT2A, NF1, and TP53, but further research is required to evaluate the potential of specific mutations in personalizing treatment strategies for MPNST. This highlights the critical importance of molecular profiling of tumors, particularly in identifying PD-L1 positivity, and serves as a basis for considering immunotherapy in treatment plans.

The first direction of research on targeted therapy was erlotinib, an inhibitor of epidermal growth factor receptor (EGFR), which is overexpressed in MPNST cell lines. Unfortunately, no activity was demonstrated in the clinical trial [50]. Other studies have been unsuccessful using dasatinib, sorafenib, imatinib, and alisertib [51,52,53,54]. Contemporary research is focused primarily on inhibitors such as pazopanib or tivozanib [22,55]. Pazopanib is an antiangiogenic agent that works by inhibiting vascular endothelial growth factor receptors (VEGFR) and platelet-derived growth factor receptor (PDGFR). These pathways are crucial for tumor angiogenesis and growth, and their inhibition is a promising strategy for combating MPNST. Another investigational agent is Selinexor, an oral exportin 1 (XPO1) inhibitor. In a retrospective analysis conducted by Al Ezzi et al., Selinexor’s efficacy was assessed in patients with unresectable or metastatic MPNST [56]. The study demonstrated a notable reduction in tumor size, of 10% to 56%. Given the limitations of this study, including a small sample size and poor data quality, further robust clinical trials are necessary to confirm these preliminary findings and to better establish selinexor’s potential therapeutic role. Future research not only in MPNST, but also other sarcomas is directed increasingly at exploring combinatorial therapeutic strategies that target multiple signaling pathways including the BCR-ABL1 TKIs imatinib, EGFR TKI erlotinib, mTOR inhibitor combined with Hsp90 inhibitor ganetespib, anti-VEGF monoclonal antibody bevacizumab, multikinase (CSF-1R) inhibitor pexidartinib, multikinase (RAF/MEK/ERK/VEGFR/PDGFR) inhibitor sorafenib and others [13,15,16,17,39,40,50,52,53,54,57,58]. Future research directions are the potential of immunotherapy, particularly anti-PD-1 agents in tumors characterized by CD274/PD-L1 amplification [59]. These targets represent a promising avenue for improving treatment responses in MPNST. Studies have shown that trametinib therapy in a patient with NF1 and MPNST shows a high efficacy of antitumor responses. Therefore, the above therapies may improve overall survival and comfort in patients with the pathology we present.

## 4. Conclusions

The treatment of MPNST in patients with NF1 is challenging due to the aggressive course of the disease, the high tendency to relapse, and the limited efficacy of systemic therapies, involving oncologists, radiologists, surgeons, geneticists, and pathologists, to ensure comprehensive patient care. The case reported in this article presents a multidisciplinary approach in both diagnosis and therapy. It includes surgical resection, radiotherapy, and a novel therapeutic approach by MEK pathway inhibitors. Surgery, even in advanced stages of the disease with the presence of metastases, can be profitable, reducing symptoms, and significantly improving the patients’ quality of life. Furthermore, the use of advanced imaging, precise molecular diagnostic, and immunotherapy, holds the potential for improving outcomes, but further research is required to validate their efficacy and integration into standard treatment protocols.

## Figures and Tables

**Table 1:** Characteristics of patients with NF1 and MPNST with lung metastases. Max, maximum; NR, not reported; M, male; F, female; D, dead; ND, not dead; No., number.

Patient No.	First Author	Sex/Age	Symptoms	Location of Primary Tumor	Lung Metastasis Size Max (cm)	Type of Metastasis Treatment Used	Overall Survival (Years)
1	Wang Y. [13]	M/40	NR	Right shoulder	1.7; 2.9	Chemotherapy	12; ND
2	Melean G. [14]	M/22	Pain in the left leg	Sciatic popliteal nerve in the left leg	NR	NR	5; ND
3	Melean G. [14]	M/24	NR	Sciatic popliteal nerve in the left leg	NR	NR	3; ND
4	Albayrak S. [15]	M/25	Loss of urinary sphincter control, paraparetic spontaneous myoclonic jerks	Upper thoracic spine	NR	NR	NR
5	Celli P. [16]	F/22	Pain, motor sphincter	Intradural C2	NR	Surgical resection	0.5; D
6	Cunha K. [17]	M/23	NR	Left lower limb	NR	Chemotherapy	0.23; D
7	Cunha K. [17]	F/23	NR	Sacrum and spine	NR	Radiotherapy	0.64; D
8	Cunha K. [17]	F/60	NR	Right lower limb	NR	Surgical resection	3.4; ND
9	Cunha K. [17]	M/24	NR	Right upper limb	NR	Chemotherapy	1.07; D
10	Cunha K. [17]	M/45	NR	Abdomen	NR	Surgical resection	1.23; D
11	Hanai U. [18]	F/59	NR	Occipital region	NR	Surgical resection	1.33; D
12	Larson K. [19]	M/60	NR	Para vertebral tumor at T7–T8	3.5	Surgical resection, Pembrolizumab treatment	3.0; ND
13	Presented case	M/24	Dyspnea, heartburn, intermittent tachycardia, right shoulder pain, expectoration of white sputum	Left lateral retroperitoneal region	22 × 13	Surgery, Trametinib treatment	NR

## Data Availability

The data presented in this study are available in this article.
